# *OsWNK9* regulates the expression of key transcription factors, phytohormonal, and transporters genes to improve salinity stress tolerance in rice

**DOI:** 10.1038/s41598-025-14775-6

**Published:** 2025-08-22

**Authors:** Yogesh Negi, Kundan Kumar

**Affiliations:** Department of Biological Sciences, Birla Institute of Technology and Science, Pilani, K K Birla Goa Campus, Zaurinagar, Sancoale, Goa, 403726 India

**Keywords:** *OsWNK9*, Rice, Salinity, Tolerance, Signaling pathways, RNA-seq, Computational biology and bioinformatics, Plant sciences

## Abstract

Rice is a staple food crop, and salinity stress severely hinders its growth and yield. Understanding the molecular mechanisms regulating salinity tolerance is essential and requires the identification and functional characterization of salt-tolerant genes to develop rice varieties with increased tolerance to salinity stress. *With No Lysine Kinases* (*WNKs*) are serine/threonine kinases involved in various abiotic stress responses. Earlier, we reported that overexpression of *OsWNK9* mitigates salinity stress in *Arabidopsis* and rice. In the present study, we used transcriptomic analysis to provide molecular insights into the tolerance mechanism exhibited by the overexpression line of *OsWNK9* (Oe-*OsWNK9)* under salinity stress. RNA-seq analysis revealed that the Oe-*OsWNK9* exhibited significant enrichment of GO terms related to biological processes, including “response to abiotic stimulus,” “regulation of protein dephosphorylation,” “protein phosphorylation,” and “cell surface receptor signaling pathways”. The cellular component GO terms were also significantly enriched with “plasmodesma,” “plasma membrane,” “extracellular space,” “apoplast,” and “cell wall” terms. The molecular function component showed enrichment of genes associated with ADP, iron, and “polysaccharide binding,” “protein dimerization activity,” and “protein phosphatase 1 binding”. The KEGG pathway enrichment plot showed enrichment of metabolic pathways, phenylpropanoid biosynthesis, and biosynthesis of secondary metabolites. Also, we observed differential regulation of key genes involved in phytohormonal transport and metabolism, ionic homeostasis, and signal transduction pathways. This study provides new insights into the dynamics of key differential functional genes and the associated transcriptional regulatory networks involved in salt stress tolerance in rice.

**Supplementary Information:**

The online version contains supplementary material available at 10.1038/s41598-025-14775-6.

## Introduction

Rice is consumed by more than 50% of the population worldwide^1,2^. Salinity stress seriously risks the agricultural productivity of rice^3^. It has been estimated that nearly 20% of the cultivated land and about 50% of irrigated areas across the globe are under salinity stress^4,5^. Salinity stress perception and tolerance response in plants can be divided into four main steps: salinity perception, early signaling, downstream signaling, and adaptive responses^6^. Notably, K^+^ ions, Ca^2+^ ions, pH, phospholipids, reactive oxygen species (ROS), and protein kinases are involved in “early signaling” followed by “downstream signaling,” which includes changes in phytohormonal and gene expression levels. The “adaptive process” encompasses growth and developmental responses, ion exclusion and sequestration, metabolism, and synthesis of compatible solutes.

Salinity stress tolerance is polygenic^7^. Upon salinity exposure, plants can activate the expression of various genes and transcription factors to regulate ion transport and extrusion and mitigate ionic toxicity^8–10^. Key components involved in ionic balance include Na^+^/H^+^ antiporter (OsSOS1), vacuolar Na^+^/H^+^ antiporters, members of the high-affinity K^+^ transporter (HKT) family, and vacuolar H^+^-translocating pyrophosphatase^11^. Several transcription factors (TFs) such as WRKY (W-box binding factor), bZIP (basic leucine zipper), MYB (Myeloblastosis), AP2/ERF (APETALA2/Ethylene responsive factor) and NAC (NAM, ATAF, CUC) TFs are known to modulate the expression of downstream genes for adaptation to salinity stress in rice^12–14^. Besides, several phytohormones such as auxins, ethylene, abscisic acid (ABA), and gibberellins (GAs) contribute to salinity tolerance in rice^15–18^.

Protein kinases are essential regulatory components of stress tolerance mechanisms^19–21^. For instance, the gene *SOS2* encodes a serine/threonine kinase involved in the Salt Overly Sensitive (SOS) stress signaling pathway to maintain ionic homeostasis and salt tolerance^22^. Class 1 Histidine kinase transporter (HKT) family members localized on the plasma membrane play an important role in salt tolerance by removing excess Na^+^ from the xylem, thereby protecting photosynthetic organs^23^. Transcription factors (TFs) are also known to be regulated by kinases. Mitogen-activated protein kinase 5 (MAPK5) is a serine/threonine kinase that targets SALT-RESPONSIVE ERF1 (SERF1), a transcription factor in rice. Overexpression of *SERF1* showed improved tolerance to salinity stress^24^. Similarly, OSBZ8 is a bZIP class of ABRE binding transcription factor, is highly expressed in salt-tolerant rice cultivars, and is activated by an SNF-1 group of serine/threonine kinase under salinity stress^25^.

*With No Lysine Kinases (WNKs)* are serine/threonine protein kinases and conserved in all life forms except yeast^26^. Studies carried out with *WNKs* in *Arabidopsis* reflect their functions in physiological processes and abiotic stress tolerance responses^27–30^. In *Glycine max*, *GmWNK1* regulates root architecture, and heterologous overexpression in *Arabidopsis* enhanced its tolerance to salt and osmotic stress^31,32^. In *Gossypium hirsutum*, the members of the WNK family (*GhWNKs*) exhibited a differential expression profile in response to stress conditions^33^. We have previously identified nine members of the rice *WNK* gene family through a genome-wide analysis^34^. *OsWNKs* display differential expression profiles during exposure to abiotic stress treatments and upon exogenous treatment with different classes of phytohormone^26,34^. Overexpression studies of *OsWNK1* in rice depicted its role in regulating circadian rhythm and abiotic stress tolerance^35^. Earlier, we reported that heterologous expression of the *WNK9* gene in *Arabidopsis* conferred increased tolerance to abiotic stressors^36–38^. *OsWNK9*-overexpression in rice exhibited strong salinity stress tolerance. The overexpression genotype showed modulation of abscisic acid (ABA) and indole-3-acetic acid (IAA) levels under salt stress. Additionally, the overexpression lines displayed enhanced levels of antioxidant enzymes and improved root growth when exposed to salinity stress^39^. These studies reflect the potential roles of *WNK* across different plant species. However, the specific regulatory roles, downstream targets, and molecular mechanisms underlying salinity stress tolerance remain insufficiently explored. While our previous studies indicate that the overexpression of *OsWNK9* enhances salt tolerance in *Arabidopsis* and rice, the overall transcriptional changes and signaling pathways orchestrated by *OsWNK9* during salt stress have not been thoroughly characterized.

We propose that *OsWNK9* improves salinity tolerance in rice by modulating specific transcription factors, influencing phytohormonal signaling, and regulating key metabolic and stress-responsive pathways. As such, the next-generation sequencing (NGS)-based RNA-Seq method is a powerful technique to identify DEGs involved in the salinity stress response that serve as valuable genetic resources for studying salinity tolerance mechanisms, also offering enormous potential towards designing salt-tolerant rice genotypes^40–44^. In this study, we used transcriptomic analysis to identify OsWNK9-regulated differentially expressed genes (DEGs) under salinity stress conditions using next-generation RNA-Seq and to examine the expression profiles of transcription factors potentially acting downstream of OsWNK9 in mediating salt stress responses. We further aimed to assess the transcript levels of genes involved in phytohormonal metabolic and signaling pathways in the *OsWNK9*-overexpressing lines. Besides, we aimed to investigate the major metabolic pathways that may contribute to enhanced salinity tolerance. Collectively these analyses were designed to discover the molecular mechanisms driving salt tolerance in the *OsWNK9*-overexpressing rice.

## Results

### Stress and cytoskeleton-related genes were highly upregulated in Oe-*OsWNK9* under salinity stress

**Fig. 1:**
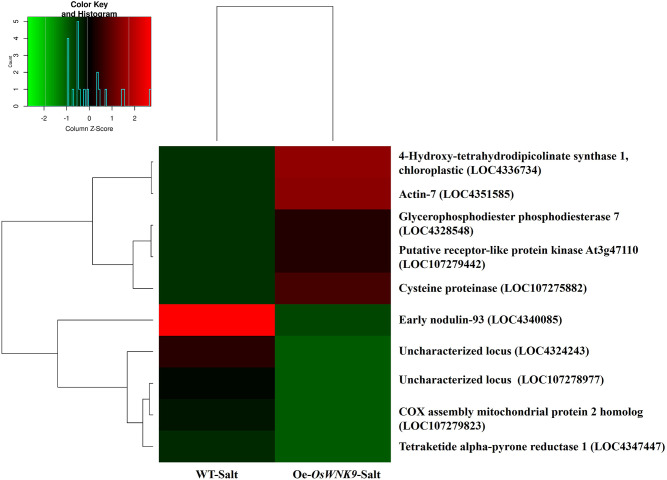
Heatmap representing differentially expressed genes (DEGs) in wild-type japonica (WT-salt) and overexpression line of *OsWNK9* (Oe-*OsWNK9*-salt) under salinity stress. Heatmap is built on taking log_2_ on FPKM values for the top 10 genes with (5 Highest FPKM values and 5 Lowest FPKM values) through Rscript. FPKM: Fragments per kilo base per million mapped reads.

To understand the changes that occur at the transcriptional level and reveal the underlying salinity tolerance in Oe-*OsWNK9*, we compared the differentially expressed genes (DEGs) in Oe-*OsWNK9* (Oe-*OsWNK9*-salt: test) and WT-JP (WT-salt: as reference) under salinity stress conditions. The summary of RNA-seq quality matrix and transcriptome assembly statistics have been summarized in Supplementary Table S2. DEGs with fold-change |log₂(FC)| > 2 and *p-*value < 0.05 were considered significant (Supplementary Fig. S1). The genes *4-Hydroxy-tetrahydrodipicolinate synthase 1* (LOC4336734), *chloroplastic; actin-7* (LOC4351585); *glycerophosphodiester phosphodiesterase GDPDL7* (LOC4328548), *putative receptor-like protein kinase At3g47110* (LOC107279442), and *cysteine proteinase* (LOC107275882) were among the top five significantly upregulated genes in the Oe-*OsWNK9* whose expressions were conversely lower in the wild-type plants under stress (Fig. 1 & Supplementary Table S3). The gene *4-Hydroxy-tetrahydrodipicolinate synthase 1* encodes a key enzyme involved in lysine biosynthesis. *Actin-7 (ACT-7)* encodes a cytoskeletal protein essential for maintaining cell structure, aiding intracellular transport, and supporting cellular functions. The enzyme GDPDL7 increases the availability of phosphate ions by catalyzing the hydrolysis of glycerophosphodiesters, which alleviates the deficiency of these ions during salinity stress. The overexpression genotype also showed increased transcript levels of RLPK and cysteine protease, which are genes linked to growth, development, and responses to abiotic stressors. These findings indicate that Oe-*OsWNK9* modulates key genes linked to cellular growth and stress adaptation, thereby enhancing salinity tolerance.

### Salinity stress induces broad upregulation of salt-responsive TF families in *OsWNK9*-overexpressing rice

**Fig. 2:**
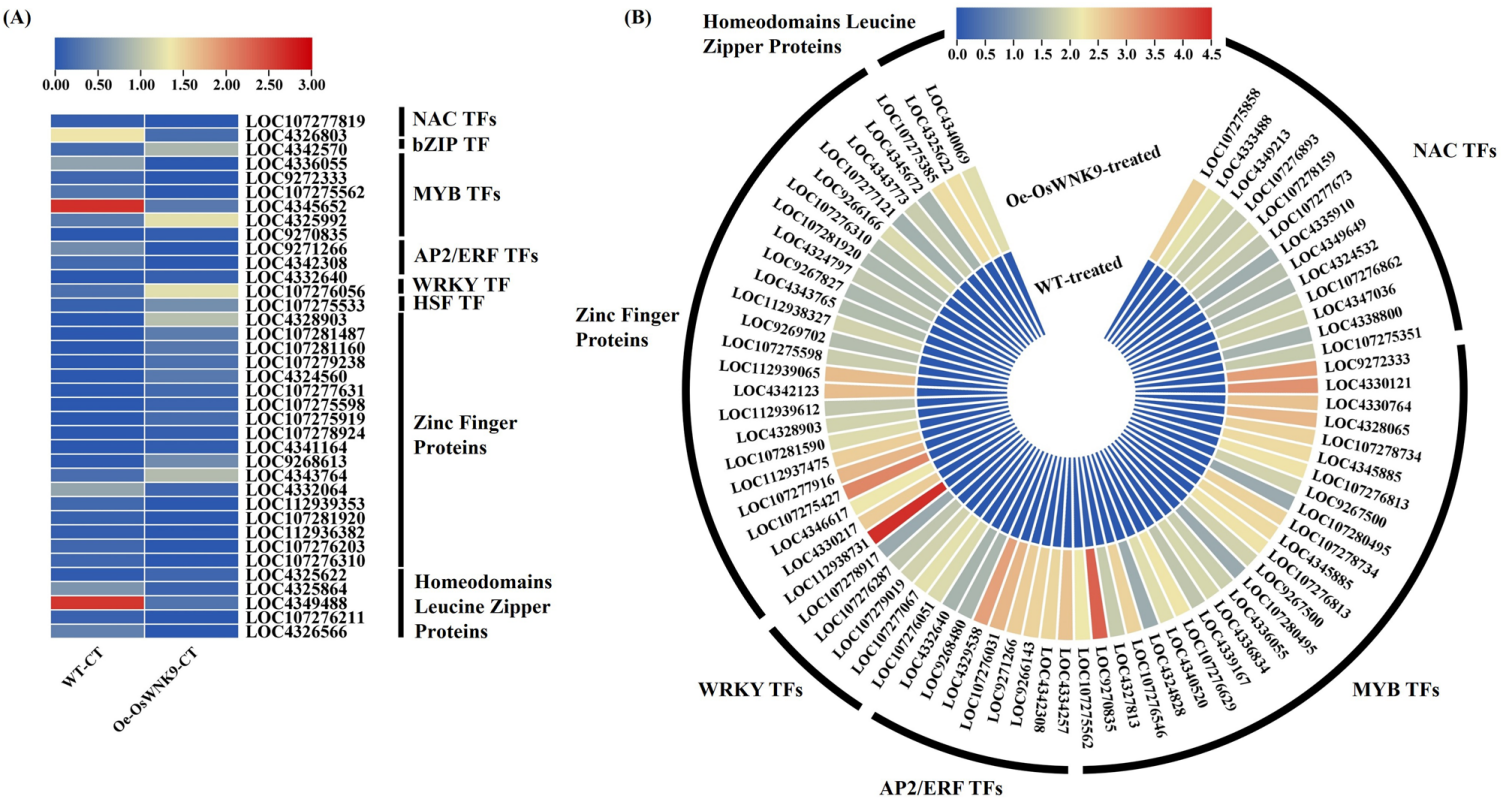
Expression patterns of transcription factors (TFs) genes. The heatmaps of expression patterns between two genotypes WT-JP and Oe-*OsWNK9* under (**A**) control and (**B**) salinity stress (150 mM NaCl).

Examination of the major salt-related TFs families, including *NAC*,* bZIP*,* MYB*,* AP2/ERF*,* WRKY*,* Zinc finger (ZF)*, and *homeodomain-leucine zippers (HD-ZIPs)*, was carried out to determine their expression patterns under control (no salinity stress) and treatment (150 mM NaCl) conditions in WT-JP and Oe-*OsWNK9*. Under control conditions, in both the compared genotypes, only a few members of salt-related TF families were observed and displayed similar expression profiles (Fig. 2 & Supplementary Table S4). However, under salinity stress, the Oe-*OsWNK9* showed drastic changes in the expression profiles of TFs, with a greater number of salt-related TF family members upregulated in the Oe-*OsWNK9* compared with WT-JP. The members of *MYB* and *ZF* TF families showed involvement of more members, followed by *NAC*,* AP2/ERF*,* WRKY*, and *HD-ZIPs*. Members of *MYB* (LOC9272333, LOC4330121, and LOC9270835); *AP2/ERF* (LOC4334257, LOC4342308, LOC9266143, LOC9271266, LOC107276031, and LOC4329538); *WRKY* (LOC107276051); *ZF* (LOC112938731) and *HD-ZIPs* (LOC4325622) TF family were highly upregulated upon salinity-treatment in the Oe-*OsWNK9*. More remarkably, these members were observed to be down-regulated in the WT-JP. The gene coexpression and protein-protein interaction (PPI) analyses also confirmed the coexpression and interaction of OsWNK9 protein with TFs (Supplementary Fig. S2 and Fig. S3). Thus, the transcription factors (TFs), particularly belonging to *NAC*,* MYB*,* AP2/ERF*,* WRKY*, and *ZF* families, were upregulated in Oe-*OsWNK9* under salinity stress.

### Expression profiles of phytohormonal-related genes in *OsWNK9*-overexpressing rice under salinity stress

**Fig. 3:**
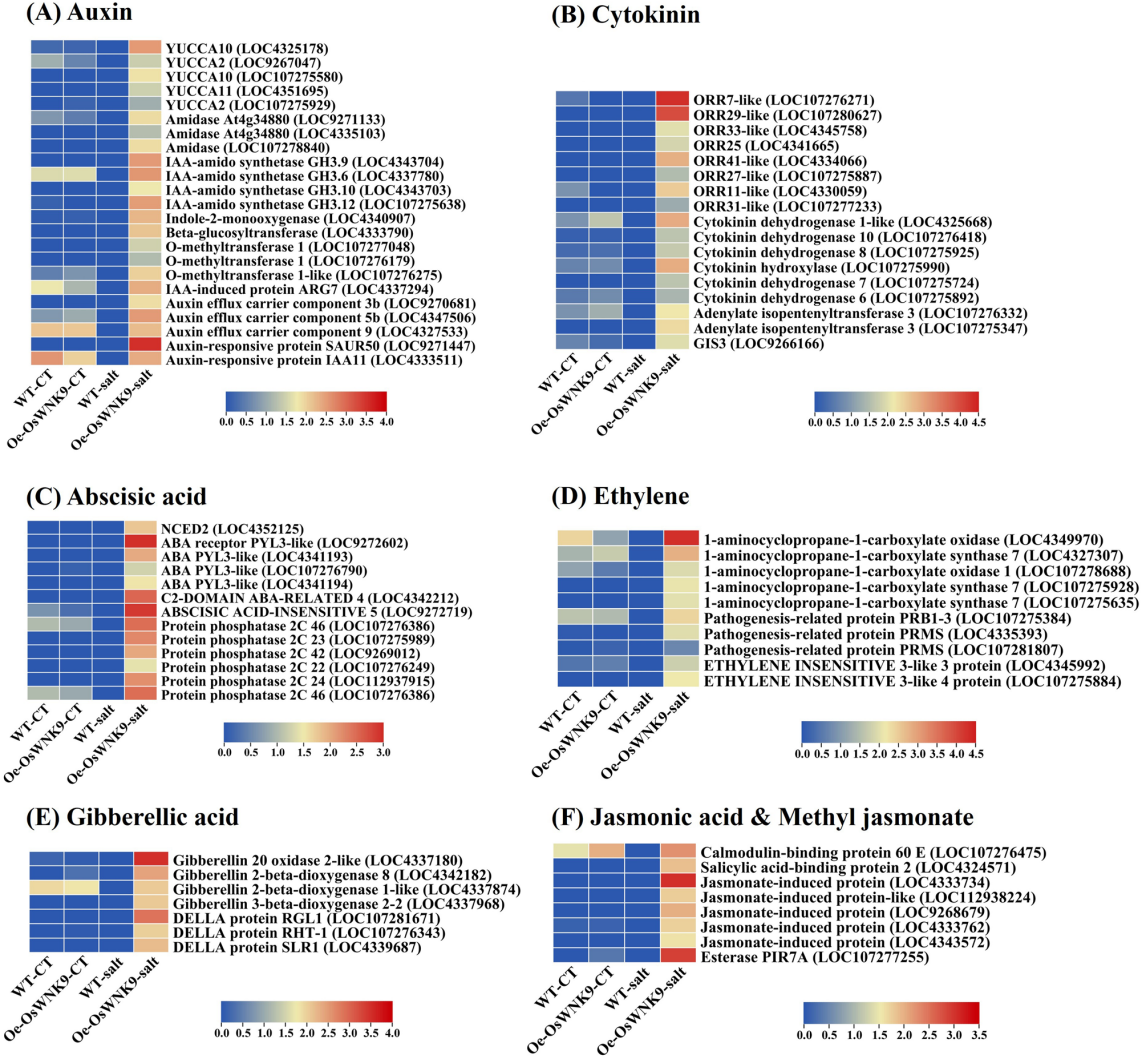
Expression profiles of phytohormone signaling and metabolism-related genes. Heatmaps illustrate the expression patterns of genes involved in phytohormone signaling and metabolism-related genes (**A**) auxin, (**B**) cytokinins, (**C**) abscisic acid (ABA), (**D**) ethylene (**E**) gibberellins and (**F**) salicylic acid (SA) and methyl jasmonic acid (Me-JA).

The highest number of upregulated differentially expressed genes (DEGs) was found to be associated with the auxin signaling pathway, followed by cytokinin, abscisic acid, and ethylene pathways. While genes involved in the gibberellic acid, jasmonic acid, and salicylic acid pathways also exhibited differential expression patterns, the number of DEGs in these pathways was lower (Fig. 3 & Supplementary Table S5). Among the auxin biosynthetic genes, *YUCCA2* (LOC9267047, LOC107275929), *YUCCA10* (LOC4325178, LOC107275580), *YUCCA11* (LOC4351695), and *indole-3-acetic acid-amido synthetases* (LOC4343704, LOC4337780, LOC4343703, LOC107275638) were upregulated in response to salinity stress in the Oe-*OsWNK9*. Additionally, *indole-2-monooxygenase* (LOC4340907) and the auxin-conjugating enzyme *indole-3-acetate beta-glucosyltransferase* (LOC4333790) showed increased expression. Genes associated with auxin metabolism, such as *amidases* (LOC9271133, LOC4335103, LOC107278840) and *indole-3-acetate O-methyltransferases* (LOC107277048, LOC107276179, LOC107276275), also displayed higher expression levels. Furthermore, components of the auxin efflux carrier, including *PIN3B* (LOC9270681), *PIN5B* (LOC4347506), and *PIN9* (LOC4327533), as well as auxin-responsive proteins such as *SAUR50* (LOC9271447), *IAA11* (LOC4333511), and the indole-3-acetic acid-induced protein ARG7 (LOC4337294), were also upregulated under salinity stress in the Oe-*OsWNK9*.

The DEGs related to cytokinin responses revealed the upregulation of the *OsRR* family of response regulators involved in the cytokinin signaling pathway. The following response regulators were identified as upregulated in the Oe-*OsWNK9*: *ORR7-like* (LOC107276271), *ORR29-like* (LOC107280627), *ORR33-like* (LOC4345758), *ORR25-like* (LOC4341665), *ORR41-like* (LOC4334066), *ORR27-like* (LOC107275887), *ORR11-like* (LOC4330059), and *ORR31-like* (LOC107277233). Additionally, the zinc finger protein *GIS3* (LOC9266166), which plays a role in cytokinin-mediated signaling, was upregulated. We observed an increased expression of *adenylate isopentenyltransferase 3*, *chloroplastic* (LOC107276332 and LOC107275347), and *cytokinin hydroxylase* (LOC107275990), both of which are key genes involved in cytokinin biosynthesis. Furthermore, genes related to cytokinin metabolism, such as *cytokinin dehydrogenase 1-like* (LOC4325668), *cytokinin dehydrogenase 6* (LOC107275892), *cytokinin dehydrogenase 7* (LOC107275724), *cytokinin dehydrogenase 10-like* (LOC107276418), and *cytokinin dehydrogenase 8* (LOC107275925), transcripts were increased under salinity stress in the Oe-*OsWNK9*.

ABA is a crucial hormone known to enhance salinity stress-mediated responses in rice. Under salinity stress, the Oe-*OsWNK9* showed upregulation of abscisic acid receptor *PYL3-like* (LOC9272602, LOC4341193, LOC107276790, and LOC4341194). The transcription factor *ABSCISIC ACID-INSENSITIVE 5* (LOC9272719) and protein *C2-DOMAIN ABA-RELATED 4* (LOC4342212) involved in ABA signaling displayed increased transcript levels. Additionally, the expression of *9-cis-epoxy carotenoid dioxygenase NCED2*, *chloroplastic-like* (LOC4352125) that catalyzes the first step of abscisic-acid biosynthesis and protein phosphatases (*PP2Cs*) (LOC107276386, LOC107275989, LOC9269012, LOC107276249, LOC112937915, and LOC107276386) were also significantly upregulated. Stress-associated *Zinc finger protein 1* (LOC107277916) and *zinc finger A20 and AN1 domain-containing stress-associated protein 10* (LOC107281568) transcripts also showed increased expression.

According to the expression patterns observed under salinity stress, the Oe-*OsWNK9* showed upregulation of ethylene biosynthetic genes: *1-aminocyclopropane-1-carboxylate oxidase* (LOC4349970), *1-aminocyclopropane-1-carboxylate oxidase 1* (LOC107278688), *1-aminocyclopropane-1-carboxylate synthase 7* (LOC4327307, LOC107275928, LOC107275635), and transcription factors: *ETHYLENE INSENSITIVE 3-like three protein* (LOC4345992), *ETHYLENE INSENSITIVE 3-like four protein* (LOC107275884) mediating expression of ethylene-responsive genes.

The DEGs related to gibberellins were involved in its biosynthesis, signaling, and metabolism. Gibberellin biosynthetic enzymes such as *gibberellin 20 oxidase 2-like* (LOC4337180) and enzymes involved in its metabolism such *as gibberellin 2-beta-dioxygenase 8* (LOC4342182), *gibberellin 2-beta-dioxygenase 1-like (*LOC4337874), *gibberellin 3-beta-dioxygenase 2–2* (LOC4337968), and gibberellin-mediated growth repressor like *DELLA protein RGL1* (LOC107281671), *DELLA protein RHT-1* (LOC107276343), and *DELLA protein SLR1* (LOC4339687) were observed to be differentially expressed in WT-JP and Oe-*OsWNK9* in response to salinity stress. Compared to other classes of phytohormones, we observed only two DEGs involved in salicylic acid pathways: *calmodulin-binding protein 60 E* (LOC107276475) and *salicylic acid-binding protein 2* (LOC4324571), and six DEGs involved in MeJA pathway. The DEGs in MeJA were *60 kDa jasmonate-induced protein* (LOC4333734, LOC112938224, LOC9268679, LOC4333762, and LOC4343572), and *probable esterase PIR7A* (LOC107277255). Thus, the phytohormonal pathways were differentially regulated under salinity stress in Oe-*OsWNK9* and WT genotypes.

### Ionic transporter genes were upregulated in Oe-*OsWNK9* under salt stress

**Fig. 4:**
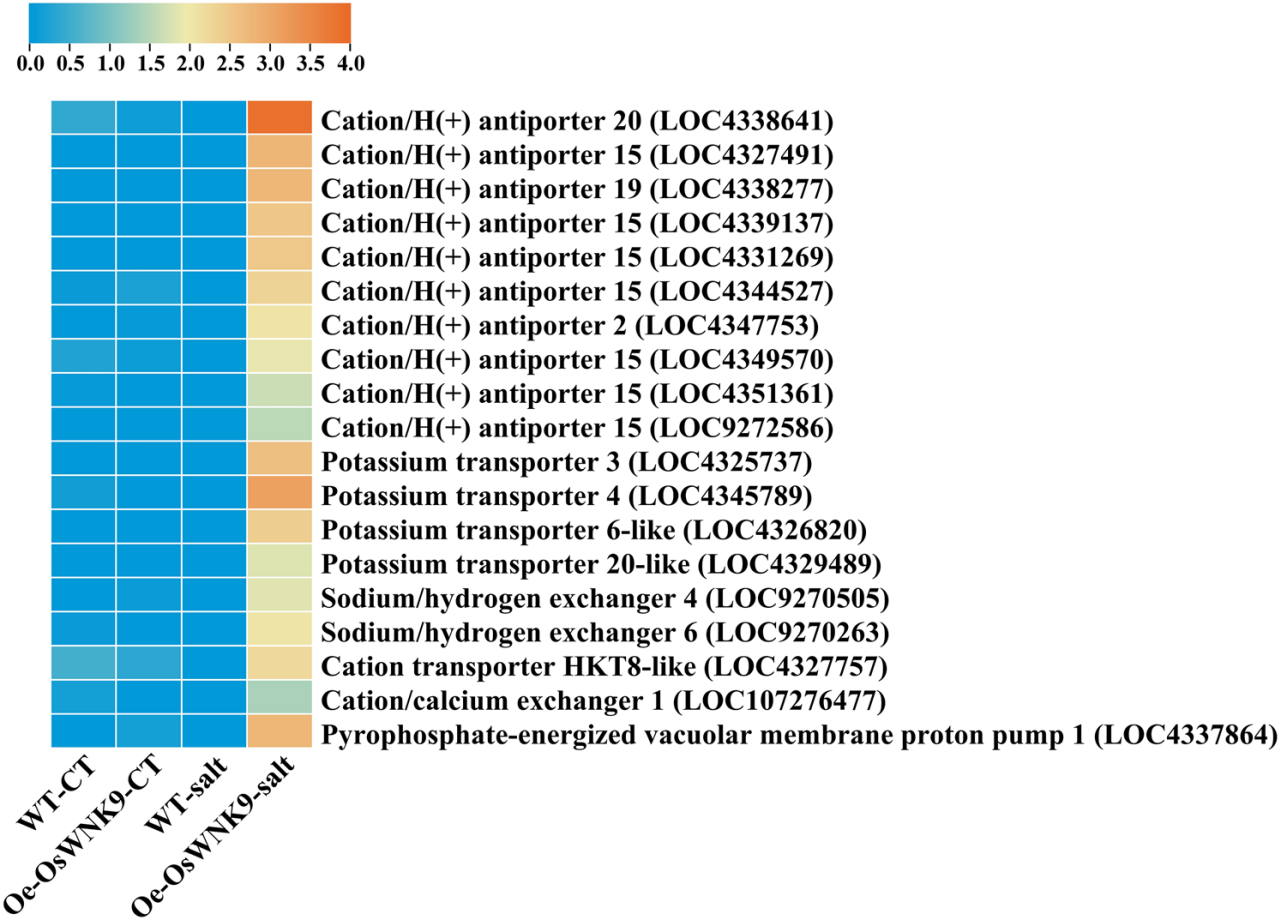
Expression patterns of transporter genes. The heatmaps represent expression patterns of transporters genes between two genotypes, WT-JP and Oe-OsWNK9, under control and salinity stress (150 mM NaCl).

Under control conditions, the compared genotypes, WT-JP and Oe-*OsWNK9*, exhibited similar expression profiles of DEGs involved in the Na^+^ ions extrusion and sequestration without significant differences in their basal expression, however, as evident from the expression profiles of transporters, salinity stress-induced expression of genes such as *cation/H*^*+*^ *antiporters*,* K*^*+*^ *transporters*,* sodium/hydrogen exchangers*,* HKT8-like*,* cation/calcium exchanger 1*,* cation-chloride cotransporter 2-like*. Among these, ten *cation/H*^*+*^ *antiporters*, four *K*^*+*^ *transporters*, two *sodium/hydrogen exchangers*, one member each of *HKT8-like*,* cation/calcium exchanger 1*, and *pyrophosphate-energized vacuolar membrane proton pump 1* showed significant upregulation in the Oe*-OsWNK9* (Fig. 4 & Supplementary Table S6) compared to the WT-JP. Besides these two genes, *SODIUM POTASSIUM ROOT DEFECTIVE 3* (LOC107276285) and *SODIUM POTASSIUM ROOT DEFECTIVE 3* (LOC4339433), were also significantly upregulated in the Oe*-OsWNK9.* Thus, from the expression profiles of transporter genes, it is evident that cation H^+ ^antiporters and K^+^ transporters were primarily upregulated under salinity stress in the *OsWNK9*-overexpression line.

### Gene ontology exhibited enrichment of signaling pathway terms under salinity stress

**Fig. 5:**
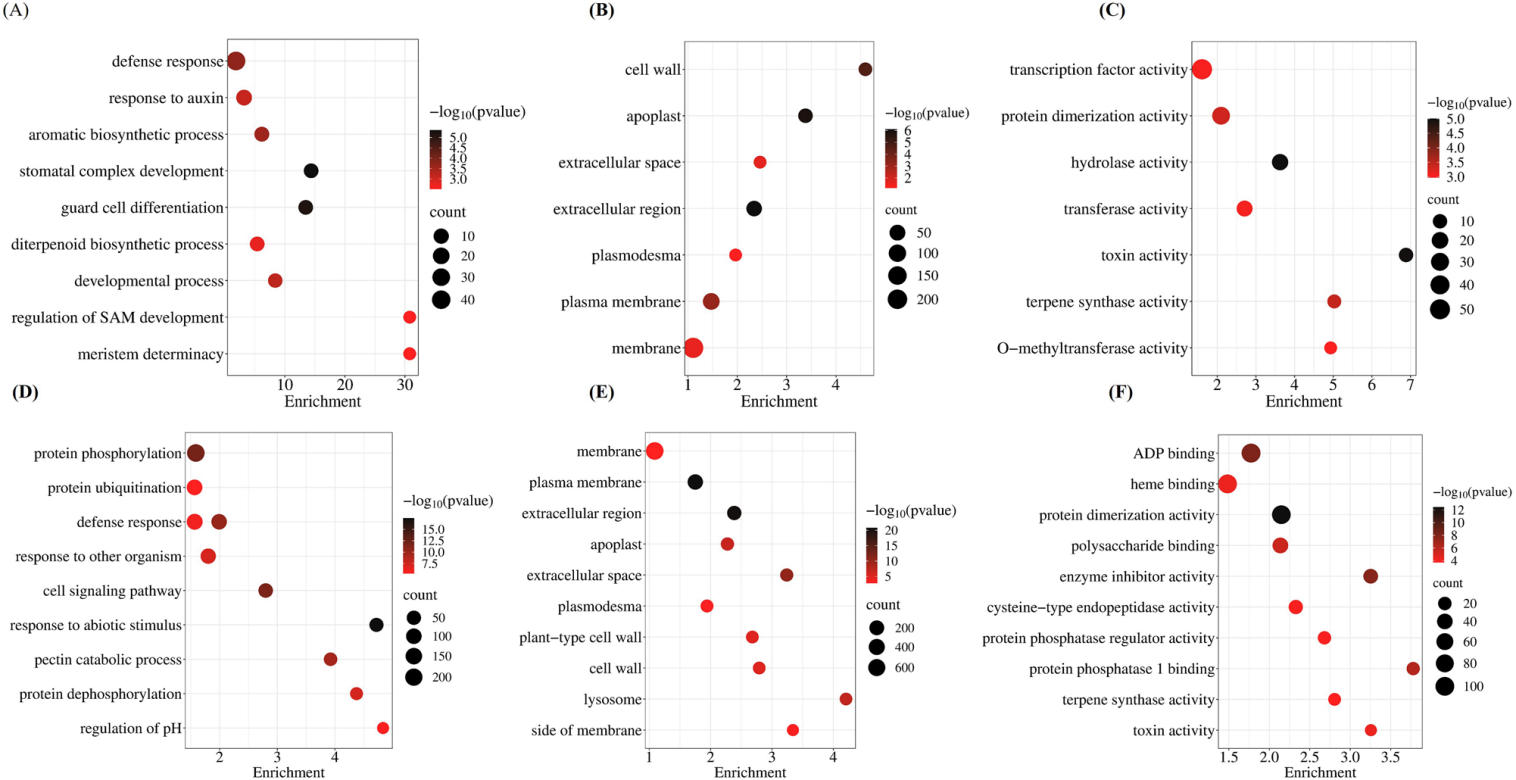
GO/KEGG function enrichment plots of Oe-*OsWNK9* compared with WT-JP. Through GO annotation, genes were categorized into Biological Process (BP), Cellular Component (CC), and Molecular Function (MF) domains. (**A**) Biological Process (**B**) Cellular Component (**C**) Molecular Function enrichment plots under control conditions and (**D**) Biological Process (**E**) Cellular Component (**F**) Molecular Function enrichment plots under salinity stress (150 mM NaCl). Enrichment dot-plot is built on taking top enrichment data sorted by p-value and then run through R script. The color of the dot represents the p-value, and the size of the dot represents the number of DEGs mapped to the referent pathway. Biological Process (BP) encompasses sets of molecular events that lead to a defined biological outcome, such as cell division or signal transduction, Cellular Component (CC) describes the locations and structures within a cell where gene products are active, such as the nucleus, and Molecular Function (MF) refers to the specific biochemical activities or functions performed by gene products, such as enzyme catalysis or receptor binding.

The GO bubble plot of comparison between control WT-JP compared with control Oe-*OsWNK9* showed 37 genes (50.0%) related to biological processes, 30 genes (40.5%) related to molecular function, and seven genes (9.5%) in the cellular component. In the control samples, GO terms were significantly enriched with biological processes, such as response to auxin (GO:0009733), aromatic compound biosynthetic process (GO:0019438), stomatal complex development (GO:0010374), guard cell development (GO:0010052), and development process (GO:0032502). The cellular component GO terms between the compared control groups were significantly enriched with membrane (GO:0016020), plasma membrane (GO:0005886), plasmodesma (GO:0009506), extracellular region (GO:0005576), apoplast (GO:0048046), and cell wall (GO:0005618). The molecular function component showed enrichment of transcription factor activity (GO:0003700), protein dimerization activity (GO:0046983), hydrolase activity (GO:0016788), transferase activity (GO:0016747), and O-methyltransferase activity (GO:0008171) (Fig. 5, upper panel & Supplementary Table S7). When salt-treated Oe-*OsWNK9* (test) was compared with salt-treated WT-JP (reference), a cumulative of 147 genes with 68 genes (46.3%) related to biological process, 60 (40.8%) to molecular function, and 19 (12.9%) to cellular component were divulged. The GO terms in biological process enrichment plot were significantly enriched with terms such as response to abiotic stimulus (GO:0009628), regulation of protein dephosphorylation (GO:0035304), protein phosphorylation (GO:0006468), cell surface receptor signalling pathway (GO:0007166), and protein ubiquitination (GO:0016567). The cellular component GO terms were also significantly enriched with terms such as plasmodesma, plasma membrane, extracellular space, apoplast and cell wall terms, similar to the enriched GO terms in control group. The molecular function component showed enrichment of genes associated with ADP binding (GO:0043531), heme binding (GO:0020037), polysaccharide binding (GO:0030247), protein dimerization activity (GO:0046983), protein phosphatase 1 binding (GO:0008157), and regulation of protein phosphatase regulator activity (GO:0019888) (Fig. 5, lower panel & Supplementary Table S7). Taken together, gene ontology (GO) showed enrichment of terms related to signal transduction pathways under salinity stress and exhibited significant upregulation of genes related to stress response, protein phosphorylation, signaling, and membrane functions.

### Secondary metabolite biosynthetic pathways were triggered during salinity stress

**Fig. 6:**
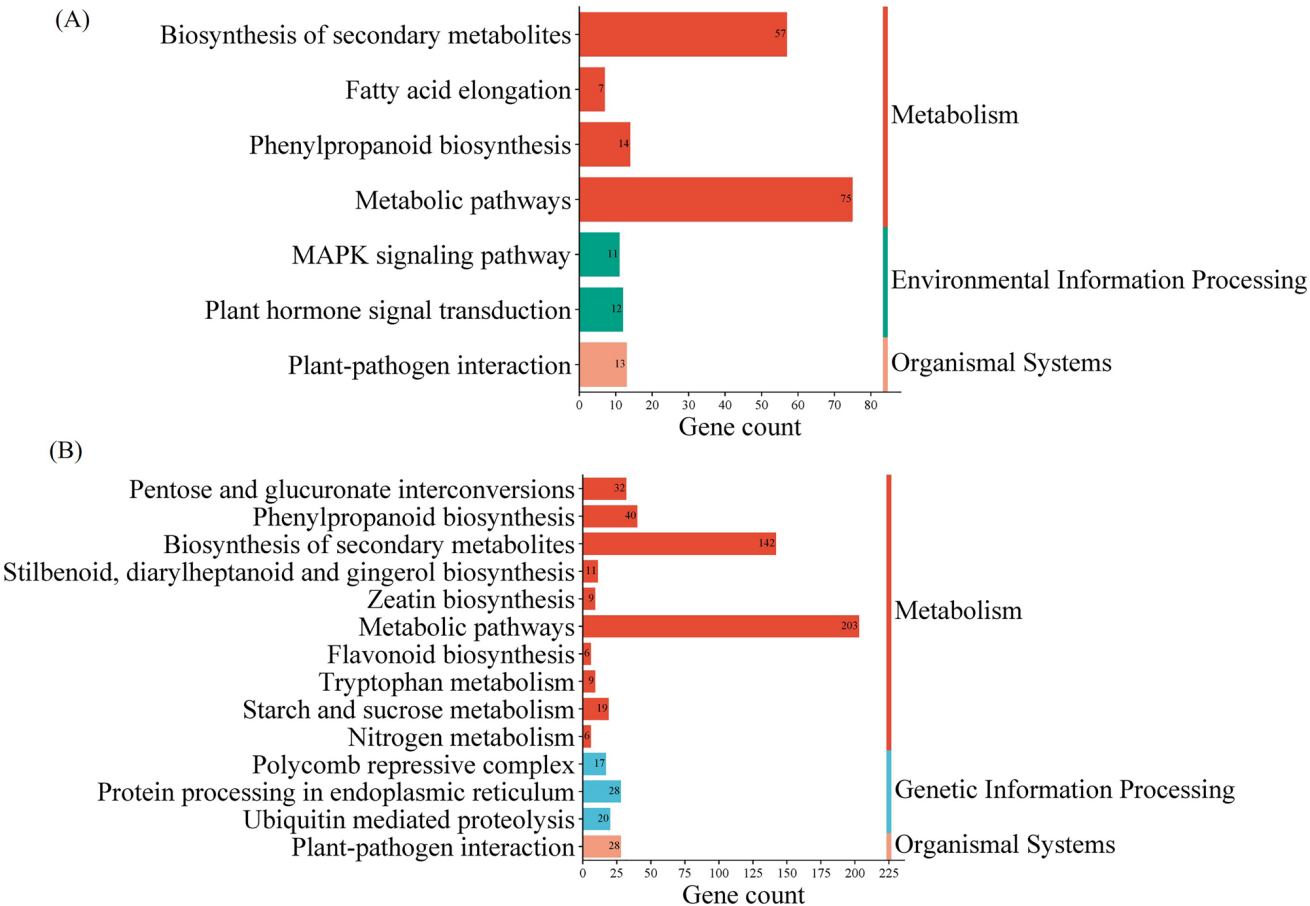
Analysis of KEGG Pathway Enrichment plots of Oe-*OsWNK9* and WT-JP for differentially expressed genes (DEGs) under (**A**) control (0 mM NaCl) and (**B**) salinity stress (150 mM NaCl). The number of significantly enriched DEGs is mentioned at the end of each bar.

The KEGG pathway enrichment plot showed enrichment of DEGs involved in metabolic pathways, phenylpropanoid biosynthesis, and biosynthesis of secondary metabolites. We noted that the number of DEGs involved in these pathways differed under control and salinity stress (Fig. 6 & Supplementary Table S8). The number of enriched DEGs was significantly higher under salinity stress than the control group. For instance, 203 DEGs were involved in metabolic processes, while only 75 DEGs were observed in the control group. Similarly, 142 DEGs were observed under salinity stress in the biosynthesis of secondary metabolites, while only 57 DEGs were present in the control group, respectively. Phenylpropane, pentoses, and glucuronates are essential building blocks of plant cell wall components. Phenylpropane monomeric units are crucial to lignin biosynthesis. In total, 40 DEGs involved in phenylpropanoid biosynthesis were enriched under salinity stress, while only 14 DEGs were annotated in the control group. Pectin is also an integral cell wall component linked with pentose and glucuronate interconversion pathways. KEGG pathway enrichment analysis showed enrichment of 32 genes related to pentose and glucuronate conversion pathways and 19 genes involved in starch and sucrose metabolism under salinity stress. We also observed 28 DEGs enriched for protein processing in the endoplasmic reticulum, 20 DEGs for ubiquitin-mediated proteolysis, and 17 DEGs for polycomb recessive protein complexes with a crucial role in gene regulation. Besides, 6 DEGs involved in flavonoid biosynthesis, 9 in tryptophan metabolism, and 6 genes in nitrogen metabolism were also enriched under salinity stress. Thus, KEGG enrichment analysis revealed that salinity stress activates genes involved in metabolism, cell wall modification, and stress-related secondary metabolite pathways.

### Validation of RNA-seq data by qRT-PCR

**Fig. 7:**
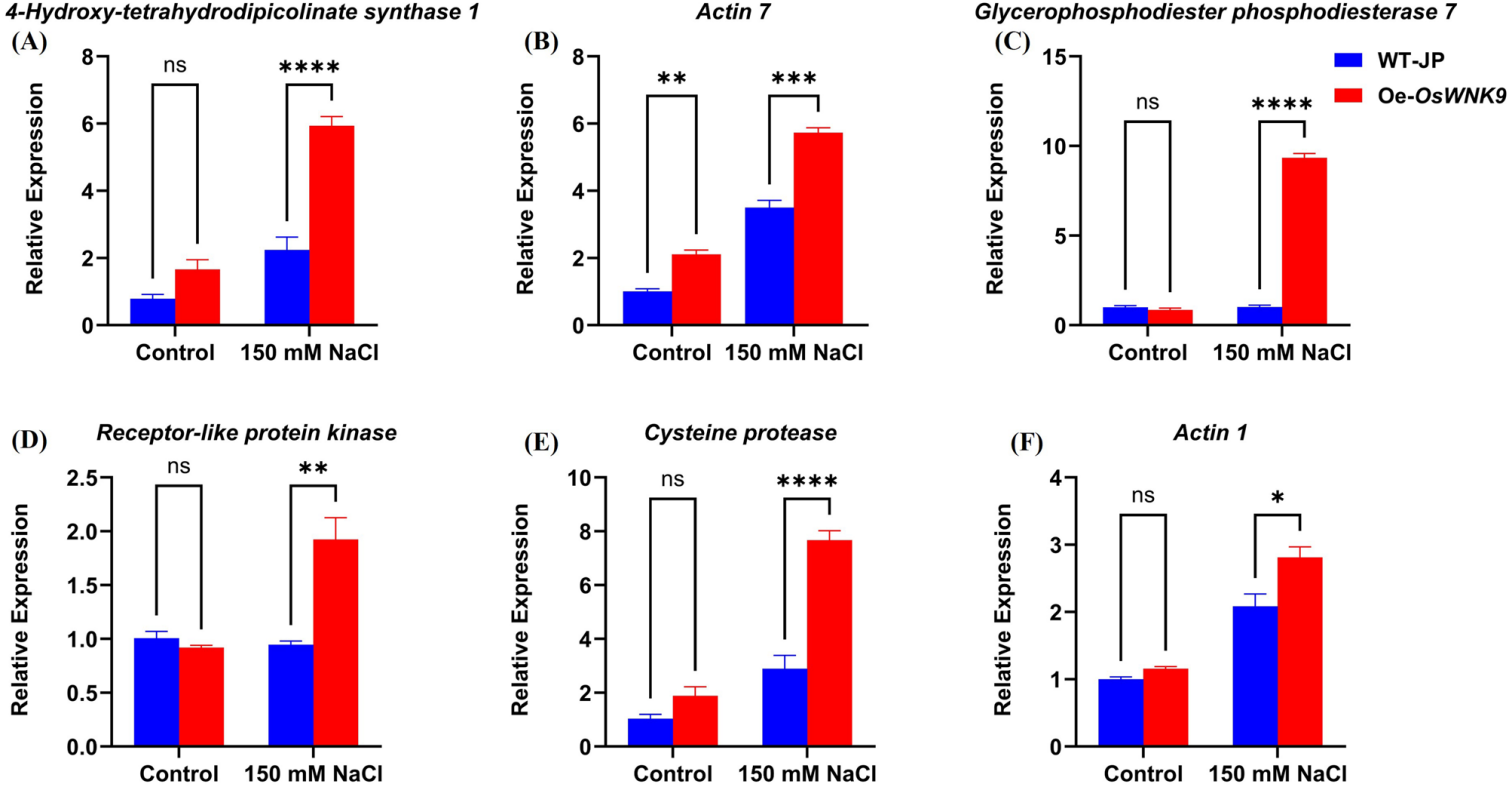
Relative expression level of the top six significantly upregulated DEGs in the Oe-*OsWNK9* compared with the WT (Japonica) genotype under control (without 150 mM NaCl) and salinity treatment (with 150 mM NaCl for 24-h). (**A**) *4-Hydroxy-tetrahydrodipicolinate synthase 1* (LOC4336734), *chloroplastic;* (**B**) *actin-7* (LOC4351585), (**C**) *glycerophosphodiester phosphodiesterase GDPDL7* (LOC4328548), (**D**) *putative receptor-like protein kinase At3g47110* (LOC107279442), (**E**) *cysteine proteinase* (LOC107275882) and (**F**) *Actin 1* (LOC4352927). Data represent mean ± SD of three biological replicates. *P < 0.0332, **P < 0.0021, ***P < 0.0002, ****P < 0.0001, by Student’s t-test.

The expression profiles of genes: *4-Hydroxy-tetrahydrodipicolinate synthase 1* (LOC4336734), *chloroplastic; actin-7* (LOC4351585); *glycerophosphodiester phosphodiesterase GDPDL7* (LOC4328548), *putative receptor-like protein kinase At3g47110* (LOC107279442), *cysteine proteinase* (LOC107275882) and *actin-1* (LOC4352927) were significantly induced under salinity stress in the Oe-*OsWNK9* compared to the WT genotype (Fig. 7). These genes were also observed to be significantly upregulated in Oe-*OsWNK9* under salinity stress as depicted by the RNA-seq analysis. Thus, qRT-PCR analysis corroborated with the RNA-seq data and reflected the possible role of these genes in salinity stress tolerance response in the Oe-*OsWNK9* genotype.

## Discussion

Rice is vulnerable to salinity stress, especially during the seedling and reproductive stages, leading to crop loss worldwide. Studies conducted through transcriptome analyses have been potentially employed to identify genes that regulate salinity tolerance^41,42^. Earlier, we reported that *OsWNK9* showed differential regulation under various abiotic stress conditions^34^. Overexpression of *OsWNK9* in *Arabidopsis* and rice showed enhanced tolerance response towards salinity stress^36,39^. The increased expression of lysine biosynthesis enzyme and salt-tolerant phenotype in the Oe-*OsWNK9* corroborates with the published report, which showed that transgenic lysine-rich rice lines exhibit increased tolerance to salinity stress^45^. In *Arabidopsis*, the *ACT7* covariant is known to regulate germination and root growth, and loss of *ACT7* caused severe inhibition of root growth under prolonged moderate-high temperatures with a drastic reduction in cellular proliferation rate and cell elongation. Further, the *act7-4* mutant of *Arabidopsis* showed altered intracellular distribution of auxin due to reduced expression of *PIN1* and *PIN2* auxin efflux carriers^46,47^. A similar observation was made when salt-treated Oe-*OsWNK9* rice lines showed increased root growth and rise in indole-3-acetic acid (IAA) content^39^. Response and adaptation to salt stress require integrating and coordinating multiple phytohormonal signaling pathways^11^. Root developmental plasticity is a hallmark of plants’ response to abiotic stress^48^. As such, the maintenance of auxin levels is considered indispensable for the growth of roots and adaptation to environmental cues^49^. Further, genes involved in auxin biosynthetic pathways are tempo-spatially regulated in response to salinity stress^50^. The Oe-*OsWNK9* also showed significant upregulation of auxin biosynthetic genes, such as *YUCCA* and *PIN* family members, under salinity stress, highlighting auxin’s role in mediating salinity stress responses. Cytokinins exert both positive and negative effects on stress tolerance^51^. We also observed that, upon salinity exposure, genes involved in cytokinin metabolism and signaling pathways, particularly from the *OsRR* family, were significantly upregulated in the overexpression line, indicating that *OsWNK9* might facilitate growth and adaptation through activation of cytokinin-mediated path significantly.

The phytohormone abscisic acid has been well-documented as the central regulator of abiotic stress tolerance response in plants^52,53^. ABA accumulation is triggered upon salinity treatment and mitigates the deleterious effects of salt stress^54,55^. Such ABA accumulation was also observed in the Oe-*OsWNK9* rice and conferred increased tolerance to salinity stress by regulating stomatal activity^39^. RNA-seq data in parallel showed that in the Oe-*OsWNK9* genotype, the abscisic acid receptor *PYL3-like*,* 9-cis-epoxy carotenoid dioxygenase NCED2*, *chloroplastic-like*, and *PP2Cs* genes were upregulated under salinity stress compared to the WT-JP. The abscisic acid receptor, *PYL*, is a positive regulator of the ABA signal transduction pathway in abiotic stress tolerance response^11^. Similarly, the ABA biosynthetic genes *NCEDs* increase ABA accumulation and enhance salinity stress tolerance^56,57^. Additionally, overexpression of *NCED4* in rice conferred tolerance to salinity and cold by ABA accumulation and ROS homeostasis^58^. Genetic analysis of a rice *PP2C* (*OsPP108*) overexpressed in *Arabidopsis* showed strong salinity and drought stress tolerance with a marked reduction in ABA sensitivity^59^. Thus, the upregulation of abscisic acid (ABA) signaling components and biosynthetic pathway observed in the Oe-*OsWNK9* similarly illustrates the crucial role of ABA in salinity stress signaling.

Hitherto, the contribution of ethylene in regulating salt tolerance remains obscured, especially in rice, and is seldom reviewed^60,61^. Besides, the role of ethylene biosynthesis and signaling components differ between *Arabidopsis* and rice concerning their salt stress response^62^. Our RNA-seq analysis showed that the ethylene biosynthetic genes and associated TFs regulating the ethylene signaling pathway in the Oe-*OsWNK9* displaying salinity tolerance were upregulated upon salinity stress treatment. Thus, the differential regulation of phytohormone-related genes in Oe-*OsWNK9* under salinity stress emphasizes the essential role of *OsWNK9* in enhancing stress resilience. Overall, these findings position *OsWNK9* as a critical component in the “early signaling response” in orchestrating “downstream phytohormonal networks” essential for “adaptive salinity tolerance responses.”

Transcription factors are vital in regulating plant growth, development, and responses to abiotic stress conditions^63^. Differential expression of transcription factor (TF) family genes regulates salinity stress tolerance in rice^64^. Overexpression of TFs such as *OsMYB6*,* OsSTAP1*,* OsNAC3*, and *OsERF106MZ* in rice showed increased tolerance to salt stress^12,13,63,65^. The differential expression of the TF family (*MYB*,* NAC*,* AP2/ERF*, and *WRKY*) and its associated members thus suggested that *OsWNK9* possibly activates critical stress-responsive pathways by targeting TFs to facilitate adaptation to salinity stress.

Salinity stress is known to disturb ionic balance, and its prolonged exposure results in ionic toxicity causing cellular injury and nutrient deficiency^12,66^. Therefore, maintaining ionic homeostasis is a significant “adaptive response” towards salinity stress tolerance^67^. The *Na*^*+*^*/H*^*+*^ *antiporter (OsSOS1)* is localized on the plasma membrane and helps exclude Na^+^ from shoots to maintain a lower cellular Na^+^/K^+^ ratio^68^. Similarly, the members of *the high-affinity K*^+^ *transporter (HKT) family are known for the* reduction of Na^+^ accrual in leaves during salinity stress^69,70^. The Na^+^/H^+^ and vacuolar H^+^-translocating inorganic pyrophosphatase OsVP1 pumps localized on the vacuolar membrane remove excess Na^+^ and K^+^ from the cytoplasm into the vacuoles and increase tolerance to salt stress^71,72^. Notably, in this study, we observed upregulation of *cation/ H*^*+*^ *antiporters*, *K*^*+*^ *transporters*,* Na*^*+*^*/H*^*+*^ *exchangers*,* high-affinity K*^*+*^ *transporter*,* cation/Ca*^*2+*^ *exchanger*, and *vacuolar membrane proton pump 1* in response to salinity stress in Oe-*OsWNK9*. The expression profile of transporter genes involved in Na^+^ ion extrusion and sequestration revealed that Oe-*OsWNK9* effectively enhances salinity tolerance through a robust ionic regulation mechanism as an effective adaptive response.

The cell wall in plants undergoes extensive modifications to deliver an adaptive response, enabling survival under stress conditions. The upregulation of lignin metabolic genes has been reported in Pokkali rice, a salt-tolerant variety^73^. The increased expression of DEGs in phenylpropanoid biosynthesis is essential for maintaining cell wall integrity and stress resilience and has also been documented in other studies^74,75^. The significant upregulation of genes involved in phenylpropanoid biosynthesis highlights the adaptive responses to counteract salinity stress in Oe-*OsWNK9*. RNA-seq analysis of Haihong 11 (H11), a salt-tolerant rice variety, showed enrichment of genes involved in carbohydrate and flavonoid metabolism and suggested their positive responses to salt stress^43^. Besides, comparative transcriptomic analysis of Xian156, a salt-tolerant rice variety with IR28 (salt-sensitive variety), showed that genes involved in nitrogen metabolism were also enriched under salinity stress^40^. GO and KEGG analyses also revealed that genes involved in flavonoid and nitrogen metabolism were also upregulated in the Oe-*OsWNK9*, and these metabolic pathways had been well documented to contribute to stress alleviation^76–79^. Gene Ontology (GO) analysis showed that Oe-*OsWNK9* activated key signaling pathways, including “response to abiotic stimulus” and “cell surface receptor signaling,” with improved regulation through transcription factors and protein phosphorylation. These findings together emphasize the multifaceted role of *OsWNK9* in regulating several metabolic and regulatory networks to enhance salinity stress tolerance in Oe-*OsWNK9* plants.

## Conclusion

**Fig. 8:**
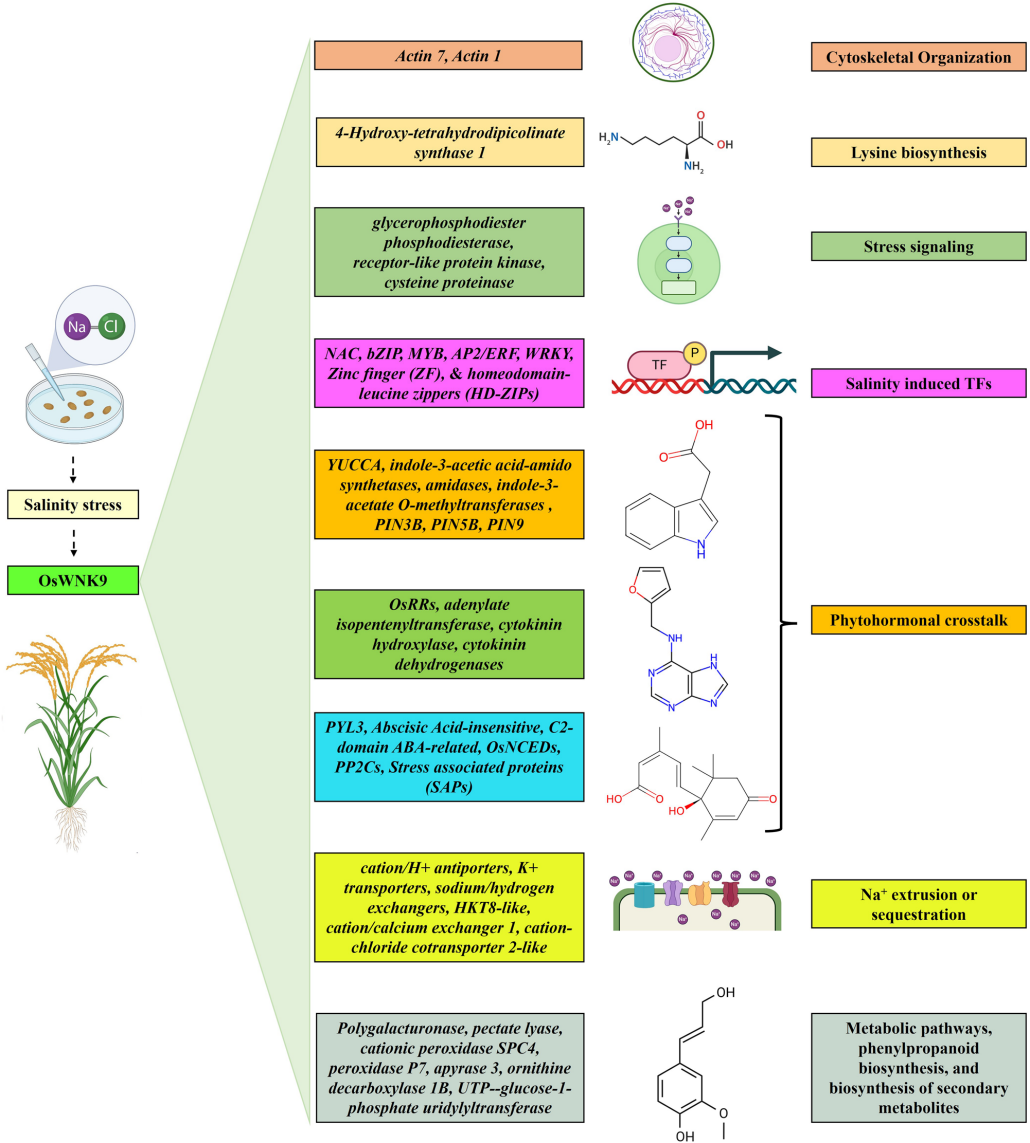
A working model of regulatory networks proposed by comparing the RNA-seq data in the *OsWNK9*-overexpression rice (Oe-*OsWNK9*) and wild-type genotypes under salinity stress. The molecular components of salinity stress tolerance response were DEGs involved in cytoskeleton organization (*actin* 7, *actin 1*); salt-responsive transcription factors (TFs): *WRKY (W-box binding factor)*,* bZIP (basic leucine zipper)*,* MYB (Myeloblastosis)*,* AP2/ERF (APETALA2/Ethylene responsive factor)* and *NAC (NAM*,* ATAF*,* CUC)*. Ionic transporters such as *cation/H*^*+*^ *antiporters*,* K*^*+*^ *transporters*,* sodium/hydrogen exchangers*,* HKT8-like*,* cation/calcium exchanger 1*,* cation-chloride cotransporter 2-like* were also differentially regulated in the Oe-*OsWNK9.* The KEGG pathway and GO analyses also indicated that extensive cell wall modifications, metabolic adjustments, and biosynthesis of secondary metabolite pathways are activated during salinity stress, which potentially underlie the salinity tolerance mechanism exhibited by the overexpression line of *OsWNK9*.

In conclusion, Our RNA-seq analysis results provided insights into the tolerance mechanism exhibited by the overexpression line of *OsWNK9* under salinity stress. Overexpression of *OsWNK9* significantly upregulated key transcription factors and phytohormonal pathways, facilitating efficient response to salinity stress. Na^+^ and K^+^ transporters increased expression emphasizes their effectiveness in maintaining ionic balance. The KEGG pathway and GO analyses indicate extensive metabolic adjustments activated during stress (Fig. 8). These findings highlight *OsWNK9* as a promising gene for improving crop resilience in saline environments.

## Materials and methods

### Plant materials and salinity stress treatment

The overexpression genotype of *OsWNK9* was previously generated in the Japonica genetic background^39^. Rice seeds of wild-type Japonica cultivar (Taipei 309) and T_2_ seeds of the overexpression line of *OsWNK9* (Oe-*OsWNK9*) were surface sterilized using 4% sodium hypochlorite (w/v) for 15 min and washed six times with autoclaved distilled water. The sterilized seeds were blotted on moistened Whatman ^TM^ filter paper number-1 and germinated for 3 days in the plant growth chamber maintained at 28 ± 2 ℃, relative humidity of about 60%, and alternating 8-h (light):16-h (dark cycle). Uniformly germinated and healthy seedlings were then selected and transferred to hydroponic culture for two weeks in greenhouse maintained at the same conditions and nourished using Hoagland’s medium supplemented with (treatment group) or without (control group) 150 mM NaCl for 24-h. Following treatment, the samples were harvested and frozen immediately in liquid nitrogen and stored at -80 ℃ until used. A total of 30 seedlings comprising 10 seedlings per condition from three independent experimental setups for each condition per genotype (WT-JP and Oe-*OsWNK9*) were sampled for each group.

### RNA isolation, quantity and quality checks, and sequencing

For RNA isolation, the standard QIAzol protocol combined QIAzol lysis with standard homogenization methods using liquid nitrogen. Briefly, 200 mg of tissue was ground to a fine powder in a mortar and pestle using liquid nitrogen and transferred to a 1.5mL tube to which 1000 µL of QIAzol Lysis Reagent was added. After adding chloroform, the homogenate was separated into aqueous and organic phases by centrifugation. RNA was precipitated from the aqueous phase by the addition of isopropanol. Subsequent purification of the RNA was done using the RNeasy Mini Kit with on-column DNase treatment as per kit instructions. The RNA was eluted using 100 µL of Nuclease-Free Water.

The RNA quality assessment was done using the RNA ScreenTape System (Catalog: 5067–5576, Agilent) in a 2200 TapeStation System (Agilent) to calculate the RINe values, based on which the RNA quality was determined. 1 µL of RNA sample was mixed with 5 µL of RNA ScreenTape Sample buffer (Catalog: 5067–5577) and heat denatured at 72 °C for 3 min, immediately placed on ice for 2 min, and the sample was loaded on the Agilent 2200 TapeStation instrument. The integrity of RNA was determined by the RNA integrity number (RINe) assigned by the software. RNA concentration was determined on the Qubit^®^ 4 Fluorometer (ThermoFisher Scientific) using the Qubit™ RNA BR Assay Kit (Catalog: Q32853, ThermoFisher Scientific). Before the sample measurement, the instrument was calibrated using the two standards provided in the kit.

Following the manufacturer’s protocol, the libraries were made using NEBNext^®^ Ultra™ II Directional RNA Library Prep Kit for Illumina^®^ (E7760L, New England BioLabs). Briefly, 500 ng of Qubit quantified total RNA was taken as input. The mRNA was isolated using Oligo(dT) beads. The isolated mRNA was then fragmented and primed using random primers and subjected to first-stand synthesis followed by second-strand synthesis. The resulting cDNA was purified using AMPure XP beads. The samples were subjected to End repair, and Illumina-specific adaptors were ligated. The adaptor-ligated product was then barcoded and subjected to 12 cycles of PCR. The samples after PCR were cleaned up using AMPure XP beads. The final enriched library was eluted in 15 µL of 0.1X TE buffer. The library concentration was determined in a Qubit 4 Fluorometer (Life Technologies) using the Qubit™ 1X dsDNA HS Assay Kits (Catalog: Q33231, ThermoFisher Scientific). The library quality assessment used the Agilent D1000 ScreenTape System in a 2200 TapeStation System (Agilent).

### Differential expression of genes

EdgeR was used to perform differential analysis. A *p*-value cut-off of 0.05 or less was used to identify the significantly expressed transcripts. A log_2_ fold-change cut-off (+ 2) was higher for upregulated transcripts and (-2) and less for downregulated transcripts. DAVID was used to identify enriched pathways in the differentially expressed genes. The figures were generated using TBTools^80^.

### Gene ontology and pathway analysis

We performed Gene Ontology (GO) annotation and enrichment analysis on the datasets for annotation. Through GO annotation, genes were categorized into Biological Process (BP), Cellular Component (CC), and Molecular Function (MF) domains. The enrichment dot plot takes the top 10 Enrichment data sorted by* p*-value and then runs through the R script. The bubble enrichment plot figures were generated using the transcriptome module in SRplot^81^. Pathway enrichment analysis was performed using the KEGG database^82–84^.

### Transcriptome sequencing data validation

Quantitative Real-Time PCR (qRT-PCR) was performed to check the reliability of the RNA-seq results. The top six highly upregulated genes under salinity stress in the overexpression genotype Oe-*OsWNK9* were selected, and primers were designed using the Primer3 tool (https://primer3.ut.ee/). The primer sets and corresponding nucleotide sequences are provided in Supplementary Table S1. qRT-PCR was performed on AriaMx Real-Time PCR System (Agilent Technologies) using SYBR green mix (2X Brilliant III SYBR^®^ Green QPCR, Agilent Technologies). The relative expression of each gene was quantified using the 2^−ΔΔCT^ method using eEF1α as endogenous control^85^.

### Statistical analysis

Data was analyzed using GraphPad Prism software (GraphPad Prism version 10). The values represent mean ± standard error (SE). Student’s t-test was used to calculate *p*-values between the means for each individual.

## Supplementary Information

Below is the link to the electronic supplementary material.

Supplementary Material 1

Supplementary Material 2

## Data Availability

The RNA-seq data have been uploaded to the NCBI SRA (Sequence Read Archive) database with reference accession PRJNA1130029. The corresponding author may be contacted if someone wants to request the data from this study.
