# Supplementary material for: OsWNK9 regulates the expression of key transcription factors, phytohormonal, and transporters genes to improve salinity stress tolerance in rice
Source: Sci Rep. 2025 Aug 22;15:30930. doi: 10.1038/s41598-025-14775-6 (PMC12373933; doi:10.1038/s41598-025-14775-6)
Supplement: Supplementary file 1 — Supplementary Material 1 [file 41598_2025_14775_MOESM1_ESM.docx]

**Supplementary Information**

**Title: *OsWNK9* regulates the expression of key transcription factors, phytohormonal, and transporters genes to improve salinity stress tolerance in rice**

Yogesh Negi, Kundan Kumar^*^

Department of Biological Sciences, Birla Institute of Technology & Science Pilani, K. K. Birla Goa Campus, Goa-403726, India

*Corresponding author: Kundan Kumar (kundan@goa.bits-pilani.ac.in)


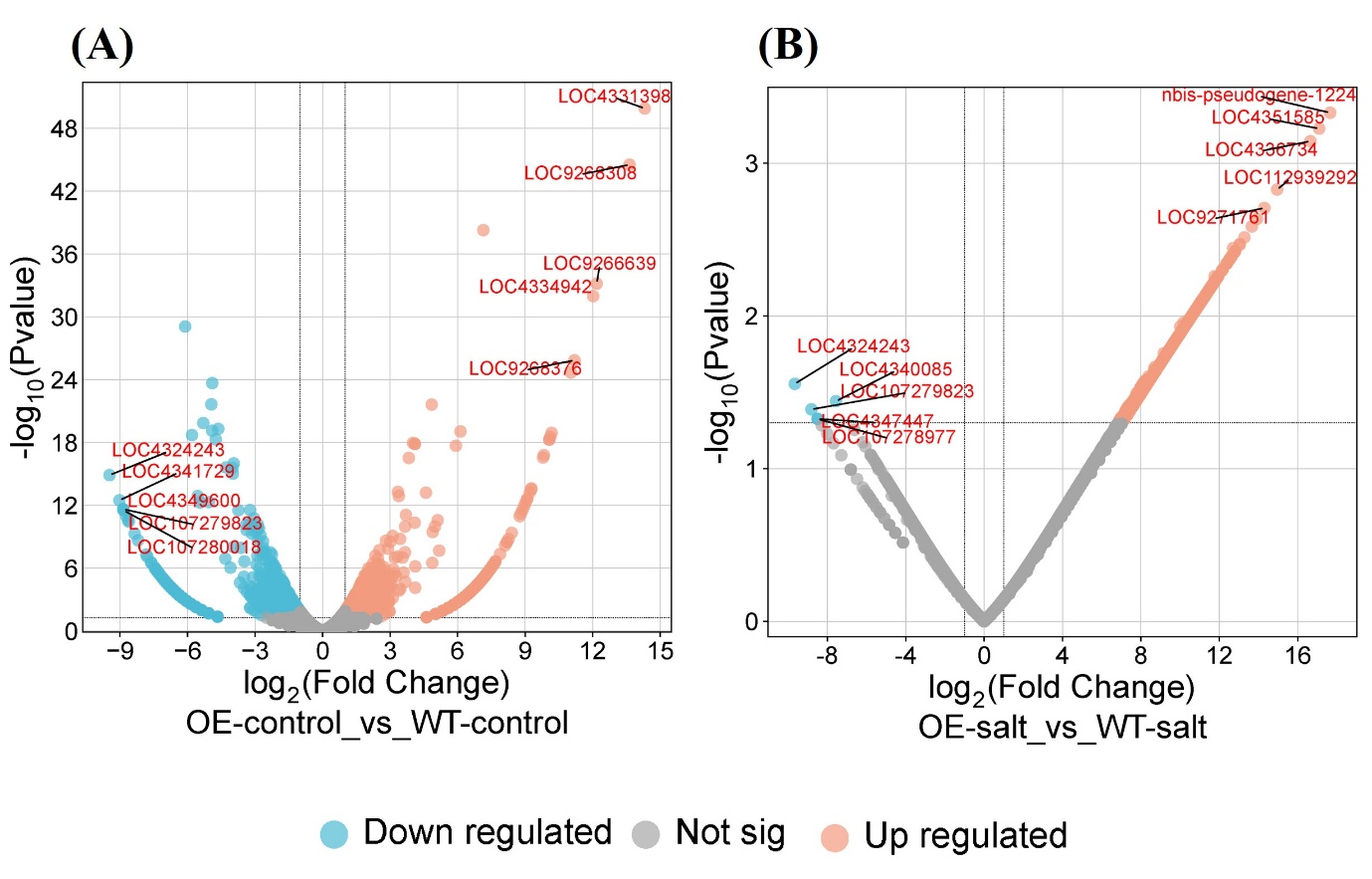
Fig. S1 Volcano plot of differentially expressed genes under (A) control (no NaCl) and (B) salinity stress (150 mM NaCl) between wild-type (WT) rice genotype and overexpression line of rice *OsWNK9* (OE). Red dots represent upregulated genes, blue dots represent downregulated genes, and gray dots represent genes that were not differentially expressed (P < 0.05). The Top 5 upregulated and Top 5 downregulated genes are labelled with the gene ID.


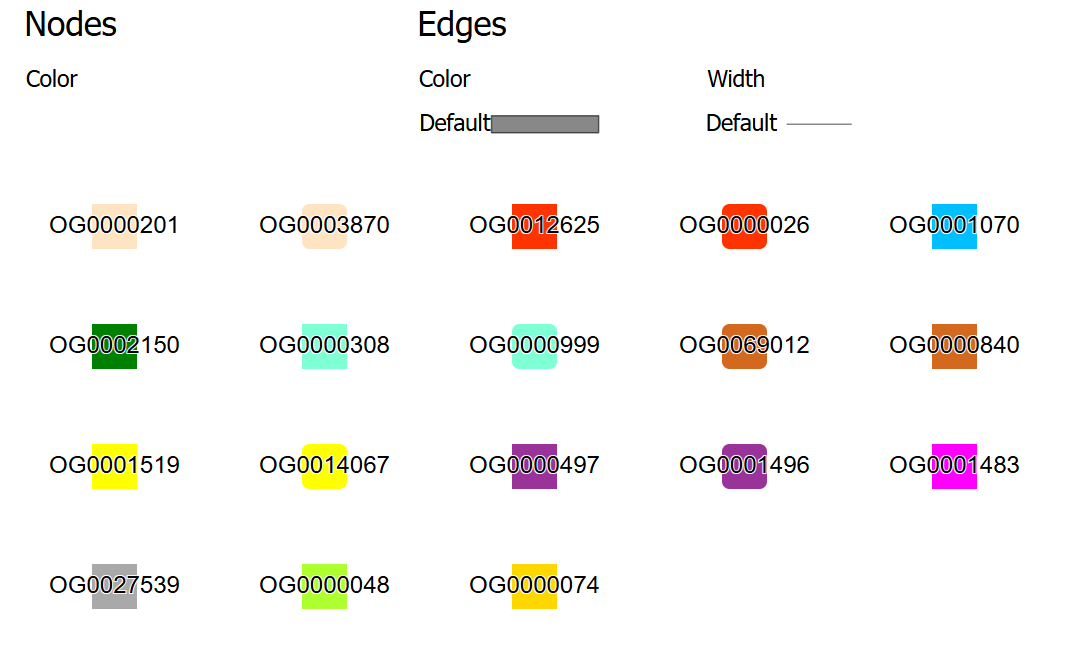

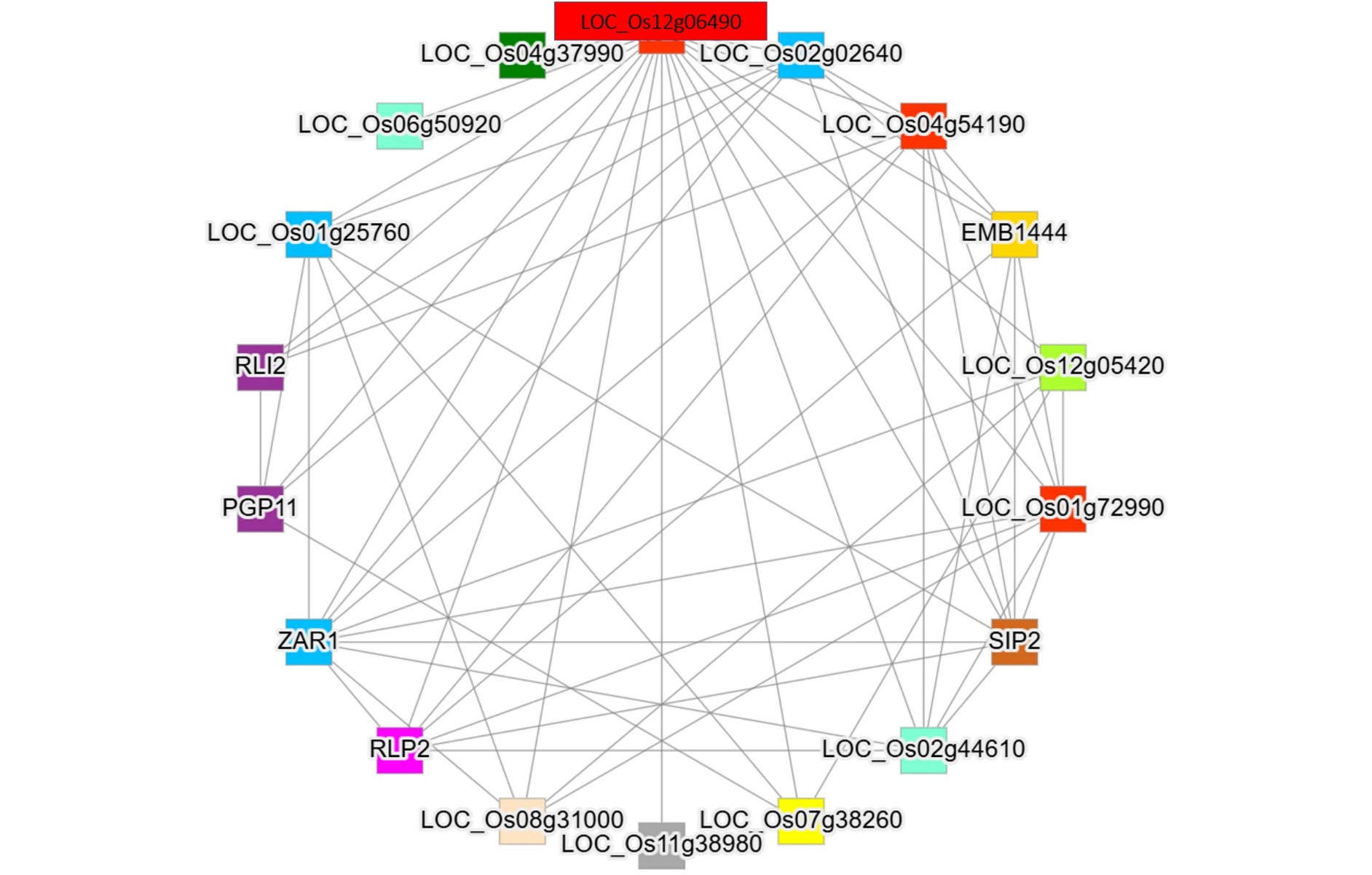
Fig. S2 Coexpression network analysis of the *OsWNK9* gene. The network includes *OsWNK9* (LOC_Os12g06490) and additional coexpressed genes identified via a co-expression network built using the conekt online tool (<https://conekt.sbs.ntu.edu.sg/custom_network/>).


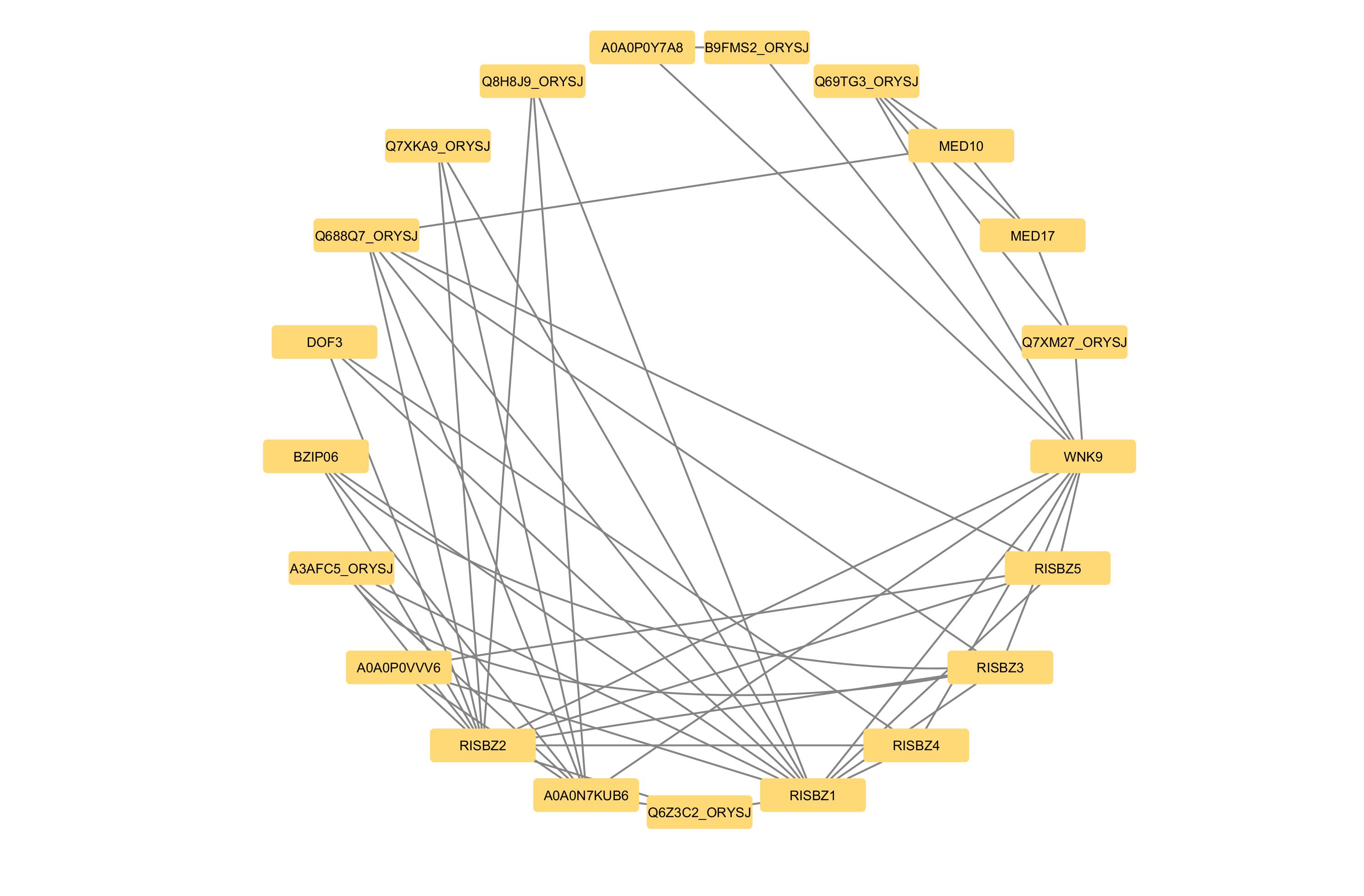
Fig. S3 Predicted protein–protein interaction (PPI) network of OsWNK9 and associated transcription factors. The network includes OsWNK9 (WNK9), bZIP family members (RISBZ1–5, BZIP06), DOF3, Mediator subunits (MED10, MED17), and additional co-regulatory proteins identified via interaction prediction. Nodes represent proteins, and edges represent predicted interactions. The PPI network was mined using STRING-DB and represented using Cytoscape.
